# Does Nice or Nasty Matter? The Intensity of Touch Modulates the Rubber Hand Illusion

**DOI:** 10.3389/fpsyg.2022.901413

**Published:** 2022-06-13

**Authors:** Letizia Della Longa, Sofia Sacchetti, Teresa Farroni, Francis McGlone

**Affiliations:** ^1^Department of Developmental Psychology and Socialisation, University of Padova, Padua, Italy; ^2^School of Psychology, Liverpool John Moores University, Liverpool, United Kingdom

**Keywords:** rubber hand illusion, body ownership, interoceptive processing, affective touch, unpleasant touch

## Abstract

Our sense of body ownership results from the ongoing integration of perceptual information coming from the different senses (i.e., multisensory integration). The Rubber Hand Illusion (RHI) has been extensively studied to investigate the malleability of body ownership through contrasting multisensory information. Indeed, during the RHI, stroking a visible rubber hand synchronously to participants’ hand hidden from sight generates the illusion of ownership of the rubber hand (embodiment) and the mis-location of participants’ hand as closer to the rubber hand (proprioceptive drift). It is well known that the RHI is optimally evoked by a pleasant stroking (affective) touch, but what of an unpleasant (painful) stroking touch – does hedonic valence matter? To this aim, participants repeated the RHI while receiving different types of touch: pleasant, painful, and neutral. Results showed, for the first time, that the subjective intensity of the tactile stimulation experienced across the different conditions modulates the strength of the proprioceptive drift. Notably, participants reported a stronger RHI (mis-placed body ownership) from stimulation rated as more intense and involving an interoceptive activation (pain and pleasantness vs. neutral). We propose that interoceptive information, regardless of the valence of the stimuli (positive or negative), are perceived as more intense and enhance, through the activation of the limbic system, multisensory integration. In the context of the RHI, this translates to a stronger illusion in terms of proprioceptive drift.

## Introduction

“Body awareness” is an umbrella term that indicates the sense that we have of our own bodies: an understanding of the parts that make up one’s body, where they are located, and how they feel (Haugstad et al., 2006; Bekker et al., 2008). In turn, awareness of one’s own body represents the foundation for the development of individual psychological identity and critically influences many interactions with the external world (Baumeister, 1999; Tsakiris et al., 2007). Body awareness largely relies on the sense of body ownership, which refers to the feeling that our body belongs to ourselves, and the localization of our body in the environment, based on the experience that our body occupies a given location in space (Serino et al., 2013). These abilities are commonly taken for granted, and largely unconscious; however, body awareness and body ownership are not constant qualities of one’s own body perception, as they are constantly influenced and updated based on ongoing multisensory information (i.e., the elaboration and integration of perceptual and emotional information coming from different sensory modalities; Blanke, 2012; Ciaunica and Fotopoulou, 2017).

The Rubber Hand Illusion (RHI; Botvinick and Cohen, 1998) is a well-established paradigm that has been used to investigate how the sense of body ownership is formed and can be modulated by the integration of multisensory information. Specifically, during the RHI, watching a rubber hand being stroked in the same place and at the same pace as the actual unseen hand is simultaneously stroked (i.e., visuo-tactile synchrony), gives rise to an illusory feeling of ownership over the rubber hand (embodiment). In addition, participants tend to shift the perceived position of their own hand as closer to the rubber hand (proprioceptive drift). The illusion results from the attempt to integrate conflicting multisensory information (visual, tactile and proprioceptive) to generate a coherent representation of the body on the basis of the available sensory information (Botvinick and Cohen, 1998; Tsakiris and Haggard, 2005). Indeed, stroking (tactile) evokes the proprioceptive feeling of one’s own hand to be displaced toward the seen (visual) rubber hand, together with the proprioceptive feeling that the rubber hand is one’s own. These two components of the RHI, proprioceptive drift and embodiment, reflect two complementary mechanisms of body awareness: self-location in space and feeling of body ownership (Serino et al., 2013). While proprioceptive drift has been typically considered an implicit correlate of the RHI, embodiment, assessed through self-report questionnaires, has been usually used as an explicit measure of the conscious experience of the illusion. However, the interplay between implicit (i.e., proprioceptive drift) and explicit (i.e., sense of embodiment) facets of the RHI is still under debate, with some evidence pointing to embodiment as a predictor of proprioceptive drift (Longo et al., 2008), and others suggesting that the two measures rely on dissociable processes of multisensory integration (Rohde et al., 2011; Abdulkarim and Ehrsson, 2016). More specifically, proprioceptive drift seems to rely on visuo-proprioceptive integration, and can therefore be elicited also in case of asynchronous visual information (i.e., asynchronous stroking); instead, embodiment seems to arise from visuo-tactile integration, and therefore it is not present in the asynchronous stroking (Rohde et al., 2011).

The efficacy and the strength of the RHI have been found to be modulated by a number of properties of the stimuli presented during the paradigm. The main principle the RHI is based on is the synchrony between the tactile sensation perceived on the real hand and the visual feedback seen on the rubber hand (Botvinick and Cohen, 1998; Tsakiris and Haggard, 2005). Indeed, the spatial and temporal congruence of the felt and seen location of the touch (i.e., synchrony) is an essential component for multisensory integration to occur (Bresciani et al., 2005). Conversely, when the touch is administered on different fingers (spatial discrepancy) or asynchronously between the real hand and the rubber hand (temporal discrepancy), the illusion is abolished (Botvinick and Cohen, 1998; Kammers et al., 2009). More specifically, a strong sensation of the RHI occurs when the temporal discrepancy is 300 ms or less, while it decreases as the delay lengthens (Shimada et al., 2014). Another factor that can modulate the strength of the illusion is the distance between the real hand and the rubber hand (Erro et al., 2020). It has been demonstrated that at a distance greater than 30 cm the illusion significantly decreases (Lloyd, 2007). Moreover, the aesthetic characteristics of the object used as the rubber hand has an impact on the efficacy of the illusion. Specifically, it has been shown that the illusion takes place only when the object resembles a realistic hand (anatomical plausibility; Tsakiris et al., 2010) in a likely range of plausible motions of an actual hand (Tsakiris and Haggard, 2005; Ide, 2013). Thus, anatomical and postural representations of the body modulate the efficacy of the RHI providing a reference model of the body for the integration of multisensory information (Tsakiris, 2010). These findings suggest that in order to be embodied as part of one’s own body, external objects need to satisfy some visual constraints to match the *a priori* semantic model of the body (Tsakiris, 2010).

Furthermore, research has begun to investigate how interoception, meaning the feelings of body internal states, may modulate the sense of body ownership (Herbert and Pollatos, 2012). Specifically, interoception is defined as the sense of the physiological condition of the body, and includes the perception of internal organ functions, muscular and visceral stimuli, hunger, thirst, pain, and pleasure (Craig, 2002). In this context, a fairly recent stream of research has focused on interoceptive processing linked to tactile stimulation. The sense of touch provides an important means to maintain a connection between the bodily self and the environment (Rochat and Striano, 2000). Tactile sensitivity is one of the first sensory modalities to develop *in utero* (Montagu, 1971) and it has the unique characteristic of providing both information from outside and from inside of the body. Tactile information is processed in terms of its *sensory-discriminative properties*, specifying information about the external object touching the skin, which is subsequently topographically mapped in somatosensory cortical areas, and also, in terms of *affective properties*, specifying the internal state of the organism (e.g., what the experience of being touched feels like affectively; Bremner et al., 2012), which is represented in para-limbic areas. This second dimension of touch, named affective touch, is mediated by a specialized system of mechanosensory afferents (C-tactile (CT) afferents) that selectively respond to gentle and slow caress-like touch. Indeed, CT afferents are preferentially activated by a limited range of tactile stimuli as they are velocity and temperature tuned to dynamic touch that resembles skin-to-skin contact between individuals (Olausson et al., 2008; Ackerley et al., 2014). Moreover, activation of CT afferents correlates with subjective feelings of pleasantness (Löken et al., 2009; Essick et al., 2010) and elicits implicit positive reactions (Pawling et al., 2017), implying that the CT system is related to a positive affective valence.

Importantly, CT afferents activate brain areas involved in interoceptive and social processing (McGlone et al., 2007), therefore suggesting that affective touch represents a fundamental link between external (cutaneous stimulation) and internal (socio-emotional responses) aspects of body perception. In particular, CT afferents project to the posterior insula, which has a crucial role for interoceptive processing, and it has also been linked to the sense of limb ownership (Tsakiris et al., 2007; Baier and Karnath, 2008). This suggests that affective touch may be critically involved in the sense of body ownership and therefore it is reasonable to hypothesize that manipulation of the affective dimension of touch may modulate the strength of the RHI. Following this hypothesis, previous studies have shown some evidence that affective touch, and the subsequent subjective feelings of pleasantness, can influence the intensity of the illusion. All studies investigating the effects of affective touch on the RHI showed that, as expected, slow CT-optimal touch was perceived as more pleasant compared to fast touch (Crucianelli et al., 2013, 2018; Lloyd et al., 2013; van Stralen et al., 2014). Furthermore, Crucianelli et al. (2013, 2018), and Lloyd et al. (2013) reported affective touch to induce higher levels of subjective embodiment during the RHI compared to a faster touch; while van Stralen et al. (2014), evidenced a positive effect of affective touch in increasing proprioceptive drift. On the basis of these results it was proposed that affective touch, and more generally interoception, may have a unique contribution to the sense of body ownership. However, other studies failed to replicate these results and therefore the role of affective touch on the RHI is still under debate (Fahey et al., 2019; Anell, 2020). Additionally, the mechanisms underlying the effects of affective touch in modulating the strength of the RHI need further investigation.

Indeed, it is yet to be determined whether results of previous studies were specific for affective touch, and therefore for an activation of the CT system, or whether they could be generalized to other interoceptive modalities. One way to address this issue is to compare the effects of affective touch on the RHI to the effects of an unpleasant/painful tactile stimulation. Indeed, pain, similarly to pleasure, involves interoceptive processing. However, while pleasant affective touch is characterized by a positive hedonic valence, unpleasant/painful touch has an opposite negative valence. Moreover, while affective touch is peripherally linked to the activation of CT-afferent, painful tactile sensations are linked to the activation of another class of c-fibers (i.e., c-nociceptors; Ellingsen, 2014). Painful stimuli have been rarely used in the RHI paradigm. Capelari et al. (2009) compared a traditional tactile condition (brush stroking) with a tactile-painful condition in which in addition to brush stroking participants were stimulated simultaneously also with a sharp pin. The results suggested that the illusion took place in both experimental conditions, moreover there was a trend for the proprioceptive drift to be greater in the tactile-painful condition (Capelari et al., 2009). However, given that tactile and painful stimulations were administered together in this study, it was not possible to drive any conclusion on the specific contribution of pain in the elicitation of the RHI. Other studies used soft vs. rough tactile stimuli during the RHI and modulated the congruence/incongruence between the visual and tactile information (i.e., the tactile stimulation was either congruent or incongruent with respect to the sensory quality of the material touching the rubber hand), with the final aim of investigating the role of perceptual expectancies (Schütz-Bosbach et al., 2009; Filippetti et al., 2019). The results of these two studies pointed in different directions with respect to the effect of (in)congruencies between the visual and tactile stimulation, suggesting that further studies are needed in order to investigate the role of top-down aspects of sensory stimulation to modulate the strength of the RHI. However, participants in both studies showed consistent discrimination of the perceived hedonic valence (pleasant vs. unpleasant) of the two stimulations, and they showed sensitivity to the RHI irrespectively of valence (Schütz-Bosbach et al., 2009; Filippetti et al., 2019). Although these studies did not aim to directly investigate the effect of pleasant vs. unpleasant stimulation, results seem to suggest that it is not the positive hedonic valence of the felt or seen touch that leads to an increased embodiment over the rubber hand. Notably, a study that manipulated both stroking velocity and qualities of material (soft vs. rough; van Stralen et al., 2014) indicated an interaction effect for the implicit measures of the RHI, showing a larger proprioceptive drift and a temperature drop only when the touch was both soft and slow. Conversely to the results of the studies reported above, these results seemed to posit the importance of a positive valence of the stimulation in strengthening the RHI. Thus, it remains an open question whether the perceived qualities (i.e., hedonic value) of the sensory stimulation may play a role in modulating the multisensory integration processes involved in the RHI, over and above the effects of visuo-tactile synchrony. Overall, previous studies indicate that CT fibers are likely to be involved in the modulation of body ownership (Crucianelli et al., 2013, 2018; Lloyd et al., 2013; van Stralen et al., 2014). However, these studies failed to elucidate whether the distinct contribution of CT fibers to the RHI is related to the perceived hedonic positive valence or to interoceptive activation similar to other sensory stimulations that involve interoceptive processes (i.e., pain). Most of the previous literature addressing similar issues manipulated the level of pleasantness of stimuli (e.g., fast vs. slow touch, or soft vs. rough material) rather than including a painful stimulation.

The present study aims to fill this gap in the literature by specifically comparing different tactile stimulations that involve interoceptive processing while having an opposite valence. To achieve this objective, participants were tested with the RHI while receiving three types of touch: pleasant, painful, and neutral. The neutral condition was added as a control condition and consisted in a tapping with no anticipated hedonic valence and not linked to interoceptive processing. Additionally, we analyzed how the subjective affective experience of the tactile manipulation modulated the strength of the RHI. To this aim, participants were asked to rate the valence (pleasantness and pain) and the intensity of the tactile stimulation in the different conditions, in line with classic theories of emotions defining emotional states in terms of activation (i.e., intensity) and quality (i.e., valence; Russell, 1980; Posner et al., 2005). Subjective rating of intensity of the tactile stimulation was paired with a measure participants’ arousal levels (skin conductance) as physiological correlate of the subjective experience (Russell, 1980; Posner et al., 2005; Mendes, 2009).

The purpose of the present study was to investigate whether different types of tactile stimulations, involving or not interoceptive processing, influenced the strength of the RHI. We hypothesized two possible results. On one side, one of the mechanisms that lead affective touch to induce a stronger RHI may be specifically related to the activation of the CT system, indicating the importance of a positive affective valence of the tactile stimulation. If this were the case, pleasant touch would be more effective than both neutral and unpleasant touch in eliciting the illusory sense of ownership over the rubber hand. On the other side, it is possible to hypothesize a more general involvement of interoceptive perception in modulating the sense of body ownership. If this were the case, pleasant and unpleasant touch would be both more effective than neutral touch in eliciting the RHI, highlighting the importance of interoceptive input in the formation of bodily representation, regardless of their hedonic valence. In support to this hypothesis, studies based on immersive virtual reality showed that vicarious pain and pleasure delivered to an avatar hand are more effective than neutral stimuli in eliciting behavioral and physiological reactivity and crucially in enhancing feelings of ownership of the virtual hand (Fusaro et al., 2016, 2019). Moreover, we measured subjective evaluation of the tactile experiences during the RHI in terms of valence (pleasantness and pain), intensity, and physiological arousal (skin conductance levels; SCLs) to investigate the contribution of these subjective dimensions in influencing the strength of the illusion, with the aim to progress current knowledge on which specific mechanisms can modulate the RHI.

## Materials and Methods

### Participants

The study was conducted at the Department of Psychology of the Liverpool John Moores University. Twenty-one young adults (10 females and 11 males) between the ages of 18 and 38 years old (mean age 24.9 years; females’ mean age 25.80, *SD* = 6.21, males’ mean age 24.09, *SD* = 5.99) were included in the study. Participants gave written consent for participation after being informed about the study aims and procedure. The local Ethical Committee of Psychological Research (Liverpool John Moores University) approved the study protocol (code of Ethical protocol 19/NSP/067), in accordance with the WMA Declaration of Helsinki, 2013. A power analysis using G*Power 3.0.10 (Faul et al., 2007), indicated that a minimum sample of 19 participants was needed to detect a medium effect (*f* = 0.25) with 80% power, using a repeated-measure design with six as the number of measurements and with alpha at 0.05 (two tailed).

### Stimuli and Procedure

**FIGURE 1:**
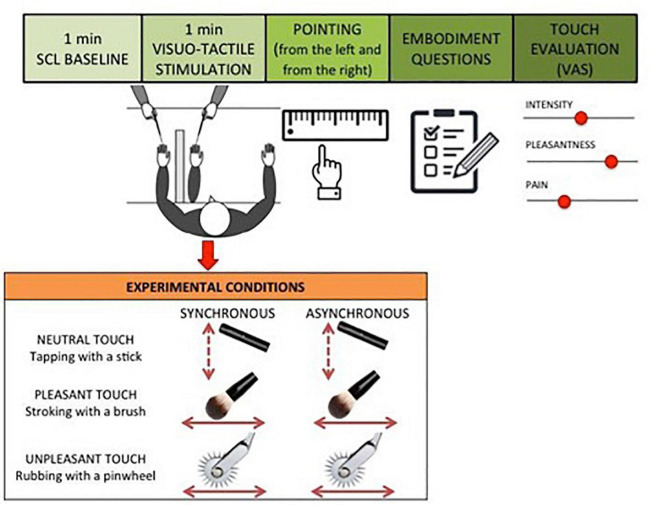
Experimental paradigm. Each participant was presented with six experimental conditions, varying the synchrony between the touch on the real hand and the visual feedback on the rubber hand (synchronous vs. asynchronous) and the type of touch (pleasant touch vs. unpleasant vs. neutral touch). The order of the experimental conditions was randomized between participants.

The experimental procedure was adapted from the RHI paradigm developed by Botvinick and Cohen (1998). Each participant was seated in front of a table directly across from the experimenter. The participant placed the left hand on the table and was asked to slide the right index finger following a ridge under the table to find the point underneath the left index finger (pointing response task) while keeping the eyes closed. To estimate each participant’s baseline ability to localize the position of their own hand, the pointing response task was repeated twice, one time starting from the left border of the ridge and one time starting from the right border of the ridge. Baseline estimation error of hand position was calculated as the difference between mean pointing response and the actual hand position. Positive values indicate a shift toward the body midline from actual hand position, whereas negative values indicate a shift away from the body midline. After that, an obstruction was placed on the table to prevent the participant from viewing their own hand and a fake rubber hand was placed on the table at a distance of 15 cm on the right from actual hand position. A black cloth was placed around the participant to cover part of both the real and the rubber arms. The trained experimenter touched the participant’s hand and the rubber hand either synchronously or asynchronously and using three different tactile stimulations: gentle stroking with a brush (pleasant touch), rubbing with a pinwheel (unpleasant touch) and tapping with a stick (neutral touch). During the stimulation, the participant was asked to closely watch the rubber hand. Each participant was presented with six RHI blocks. In each block, 1 min of baseline skin conductance level (SCL) was recorded before the administration of the visual tactile stimulation in order to measure changes in the physiological arousal in response to the different tactile conditions. Then the experimenter administered the tactile stimulation for 1 min. The tactile stimulation was manipulated between blocks, varying the synchrony between the touch on the real hand and the visual feedback on the rubber hand (synchronous – same time and same position – vs. asynchronous – different time and different position); and the type of touch (pleasant touch – stroking with a brush- vs. unpleasant – rubbing with a pinwheel – vs. neutral touch – tapping with a stick). The order of the experimental conditions was randomized between participants. As in the baseline, the same pointing response task was administered following each RHI trial. Therefore, the participant pointed two times for each experimental condition. To measure the extent to which the proprioceptive perceived position of one’s own hand was influenced by incongruent multisensory signals (induction phase of the RHI), proprioceptive drift was calculated by subtracting the mean baseline pointing response from the mean test pointing response for each experimental condition. After each RHI block, the participant was asked to complete the Embodiment Questionnaire (Botvinick and Cohen, 1998), which assesses the subjective experience of the illusion through 9 items. Each item reflects an unusual perceptual experience that may arise as a result of the RHI. For each statement participants are asked to report the level of agreement on a 7-point Likert scale ranging from −3 (strongly disagree) to +3 (strongly agree). Different scoring methods have been used in RHI research, with some studies analyzing the results of each item individually and others reporting a total score using the responses to all items. In the present study, an index score has been calculated based on the first three items, which are considered to most consistently and accurately reflect the RHI experience (Kaplan et al., 2014). Additionally, participants were asked to evaluate the tactile experience using a visual analog scale (VAS) based on two dimensions: the valence and the intensity (Bertheaux et al., 2020). Specifically, the valence corresponds to the hedonic nature of information on a continuum ranging from pleasant (+10) to unpleasant (−10). Moreover, subjective pain was measured through a VAS scale where 0 was considered to be not at all painful and 100 was considered to be extremely painful. The intensity refers to the level of activation provoked by tactile experience on a continuum ranging from the least intense sensations (0) to the most intense sensations (100; Figure 1). Finally, participants were asked to fill in questionnaires to measure individual tendencies to focus on normal, non-emotive body processes (e.g., thirst, hunger, fatigue, temperature changes; Body Awareness Questionnaire, BAQ; Shields et al., 1989), to self-monitoring and focus on pain sensations (Pain Awareness and Vigilance Questionnaire, PVAQ; McCracken, 1997) and to avoid or dislike social touch (Social Touch Questionnaire, STQ; Wilhelm et al., 2001). However, these measures were not included in the analyses of this study as they are not related to the research questions. For detailed description of Questionnaires see Supplementary Materials.

### Electrophysiological Data Recording and Processing

Electrodermal Activity (EDA) was recorded by means of two electrodes applied on the index and middle finger of the participant’s stimulated hand with each. The two electrodes were connected to a physiological monitoring device (Biopac MP30B-CE), which in turn was connected to a computer that recorded the physiological activity through the Biopac Student Lab Pro 3.7 software. The software was programed to filter the EDA signal in real time with a band-pass of 0–35 Hz. EDA data were recorded continuously during each of the six RHI blocks (i.e., experimental conditions). For each block a 1 min baseline recording was obtained prior to the administration of the RHI. Each recording was visually inspected for artifacts, which were manually removed using Biopac (MP150) Systems. Skin Conductance Levels (SCLs; corresponding to tonic electrodermal activity of the skin) were then extrapolated from the EDA data averaging across the EDA signal in each baseline and experimental recording. SCLs in each experimental condition were then corrected by the corresponding baseline value, giving six baseline-corrected SCLs values for each participant. SCLs have been used as an index of physiological arousal.

### Statistical Analysis

All statistical analyses were performed using R, a software environment for statistical computing and graphics (R Core Team, 2016). More specifically, to carry out mixed models we used “lmer” from the “lme4” package (Bates et al., 2015). In order to compute R−squared for the models, we used “r.squaredGLMM” from MuMIn package (Barton, 2009), which takes into account the marginal R−squared (associated with fixed effects) and the conditional one (associated with fixed effects plus random effects). The *p*−value was also calculated using the “lmerTest” package (Kuznetsova et al., 2017). The choice of using a mixed-effects model approach was determined by the possibility to take into account fixed effects, which are parameters associated with an entire population as they are directly controlled by the researcher, and random effects, which are associated with individual experimental units randomly drawn from population (Gelman and Hill, 2007; Baayen et al., 2008). Specifically, Akaike information criterion (AIC; Akaike, 1973) and Bayesian information criterion (BIC) have been used to compare a set of models fitted to the same data (McElreath, 2016). The model that produces the lowest AIC and BIC values is the most plausible (Hooper et al., 2008). Moreover, we compute Anova using the “car” package and contrasts using the “emmeans” package to compare each type of touch against every other type of touch. Finally, in order to explore the possible relationship between individual differences (in terms of attention to body internal states, pain sensibility and attitude toward social touch) and susceptibility to the RHI, we carried out correlations using the “rcorr” function, which returns both the correlation coefficient and the significance level, and the “corrplot” function, which returns a graphical representation of the correlational matrix. Complete graphs and tables of correlation coefficients and associated *p*-values can be found in Supplementary Materials.

## Results

### Subjective Evaluation of Tactile Stimulation

In order to check whether the tactile stimulations were effective in eliciting the expected perceptive sensations, we analyzed the participants’ subjective experience based on three self-reported scales: pleasantness and feeling of pain, which reflect the valence of the stimulation, and the perceived intensity, which reflects the level of activation. To analyze the subjective ratings we used a mixed-effect model approach. Four nested mixed-effects models were tested. In each model, the subjective rating of the tactile experience was the dependent variable. The null model (Model 0) included only the random effect of Participants; the first (Model 1) included Synchrony (2 levels; synchronous visual-tactile stimulation vs. asynchronous visual-tactile stimulation) as fixed factor and Participants as random factor; the second (Model 2) included also the Type of Touch (3 levels; stroking with a brush vs. rubbing with a pinwheel vs. tapping with a stick) as fixed factor; the third (Model 3) added the interaction between Synchrony and Type of Touch. Tables reporting the specification of model comparison can be found in Supplementary Materials.

#### Pleasantness

**FIGURE 2:**
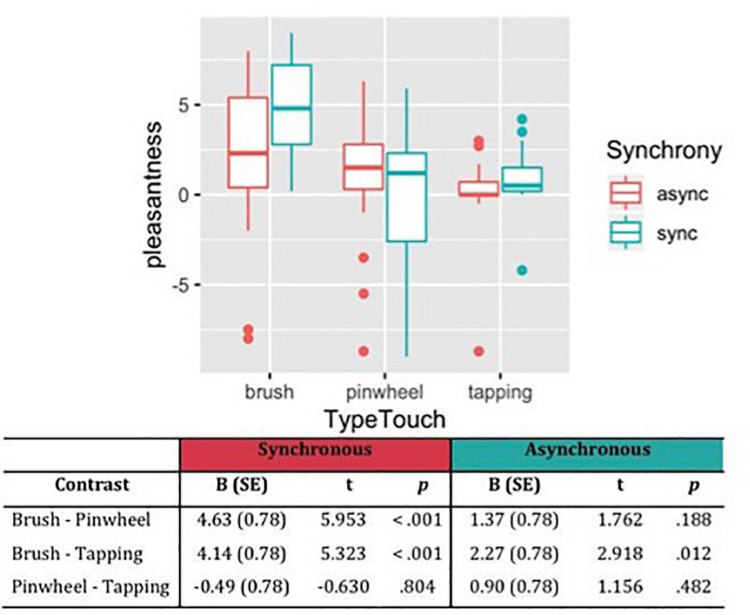
Subjective pleasantness of tactile stimulations measured on VAS scale from –10 (very unpleasant) to +10 (very pleasant). Contrasts between different types of touch have been reported, showing that brush stroking was perceived as the most pleasant stimulation, in particular in the synchronous condition.

Considering the subjective ratings of pleasantness, the likelihood ratio test showed that Model 3 was the best at predicting the collected data and included the factors Synchrony, Type of Touch and their interaction (descriptive statistics and model comparison are reported in Supplementary Tables 1,2). The model explained 21% of the variance. Anova revealed a main effect of Synchrony [χ^2^(1) = 4.04, *p* = 0.044] indicated that synchronous stimulation was perceived as more pleasant compared to asynchronous stimulation. Moreover a main effect of Type of touch [χ^2^(2) = 42.57, *p* < 0.001] and the interaction effect between Type of touch and Synchrony emerged [χ^2^(2) = 8.85, *p* = 0.012]. Specifically, contrast revealed that brush stroking is perceived as more pleasant than touch with the pinwheel and tapping, and such difference is enhanced when the tactile stimulation on the participant’s hand was administered synchronously in respect to the visual feedback on the rubber hand. On the contrary, no difference emerged between tactile stimulation with the pinwheel and tapping (Figure 2).

#### Pain

**FIGURE 3:**
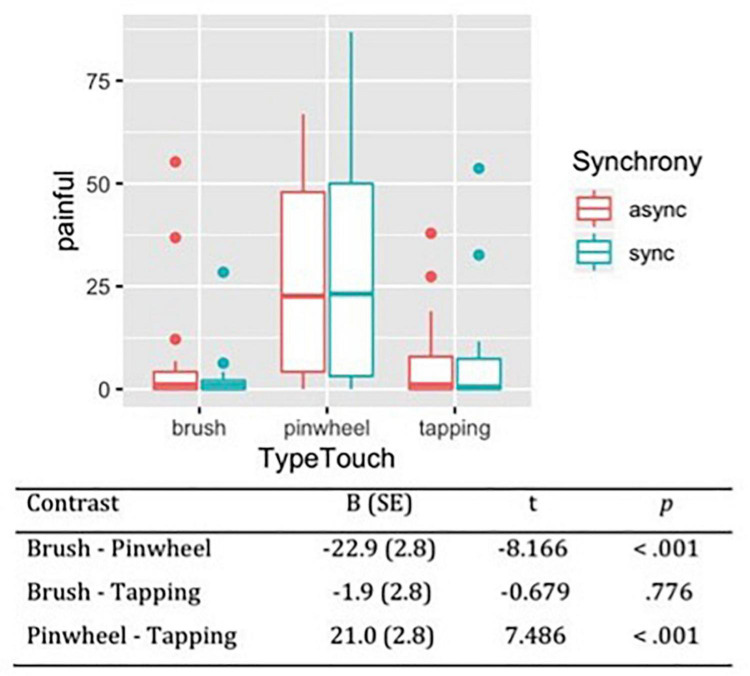
Subjective pleasantness of tactile stimulations measured on VAS scale from 0 (not at all painful) to 100 (extremely painful). Contrasts between different types of touch have been reported, showing that rubbing with a pinwheel was perceived as the most painful stimulation.

Considering the subjective sensation of pain during the tactile stimulations, the likelihood ratio test showed that Model 2 was the best at predicting the collected data and included the factors Synchrony and Type of Touch (descriptive statistics and model comparison are reported in Supplementary Tables 3,4). The model explained 26% of the variance. Avova revealed a main effect of Type of touch [χ^2^(2) = 83.10, *p* < 0.001]. Contrasts revealed that the tactile stimulation with the pinwheel was perceived as more painful (*M* = 27.47, *SE* = 3.3) than brush stroking (*M* = 4.57, *SE* = 3.3) and tapping (*M* = 6.48, *SE* = 3.3). On the contrary, no difference emerged between tactile stimulation with the brush and tapping (Figure 3).

#### Intensity

**FIGURE 4:**
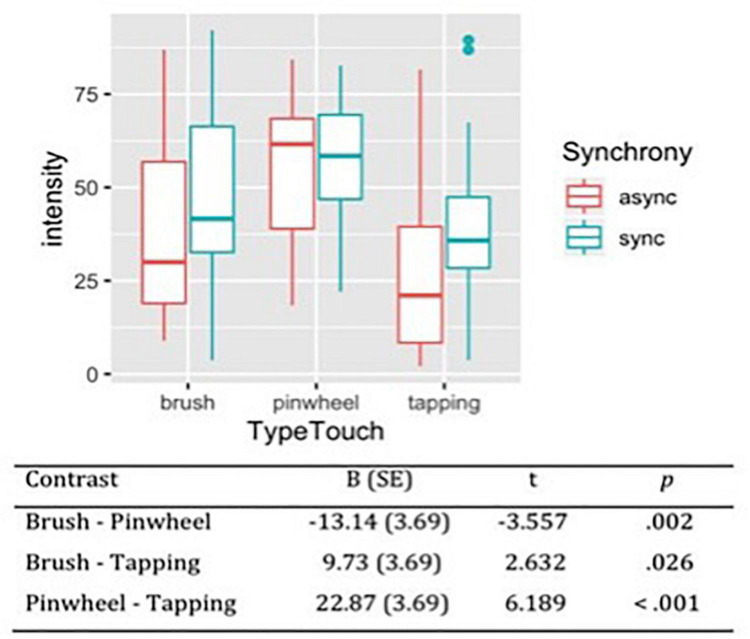
Subjective intensity of tactile stimulations measured on VAS scale from 0 (least intense) to 100 (most intense). Contrasts between different types of touch have been reported, showing that the perceived intensity of the tactile stimulation was maximal for rubbing with a pinwheel, intermediate for stroking with a brush and minimal for tapping with a stick.

Considering the subjective intensity of the tactile stimulations, the likelihood ratio test showed that Model 2 was the best at predicting the collected data and included the factors Synchrony and Type of Touch (descriptive statistics and model comparison are reported in Supplementary Tables 5,6). The model explained 19% of the variance. Anova revealed a main effect of Synchrony [χ^2^(1) = 7.65, *p* = 0.006] and Type of touch [χ^2^(2) = 38.92, *p* < 0.001]. These results indicate that synchronous stimulation was perceived as more intense compared to asynchronous stimulation. Moreover, contrast revealed that the three types of touch differed between each other in respect to the subjective intensity reported by participants. Specifically, pinwheel was reported as the most intense stimulation (*M* = 53.9, *SE* = 3.79) and tapping as the least intense (*M* = 31.0, *SE* = 3.79; Figure 4).

### Skin Conductance Level

**FIGURE 5:**
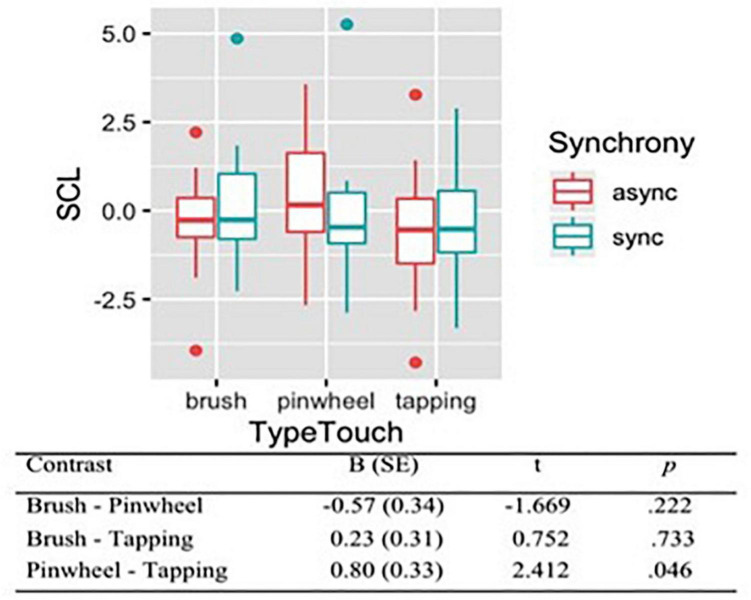
Skin Conductance Levels (SCL) during tactile stimulations. Contrasts between different types of touch have been reported, showing that rubbing with a pinwheel produced an increased of SCL on the contrary of brush stroking and tapping.

To evaluate the level of arousal during different conditions of the RHI, we analyzed the skin conductance level (SCL), which was the dependent variable of all models. A set of four nested mixed-effects models were tested including the same factors used to analyze subjective evaluations of tactile experience. Moreover we wanted to control whether the subjective valence and intensity of the touch influenced SCL, thus we tested three additional models, including self-reported pleasantness (Model 4) and painful sensation (Model 5) and intensity (Model 6). The likelihood ratio test showed that the Model 5 was the best at predicting the collected data (descriptive statistics and model comparison are reported in Supplementary Tables 7,8). The model explained 10% of the variance. Anova revealed a main effect of Type of touch [χ^2^(2) = 6.00, *p* = 0.050]. Contrasts revealed that the tactile stimulation with the pinwheel produced an increase of SCL (*M* = 0.24, *SE* = 0.30). On the contrary, brush stroking (*M* = −0.33, *SE* = 0.29) and tapping (*M* = −0.56, *SE* = 0.29) elicited a decrease of SCL (Figure 5).

### Subjective Embodiment

**FIGURE 6:**
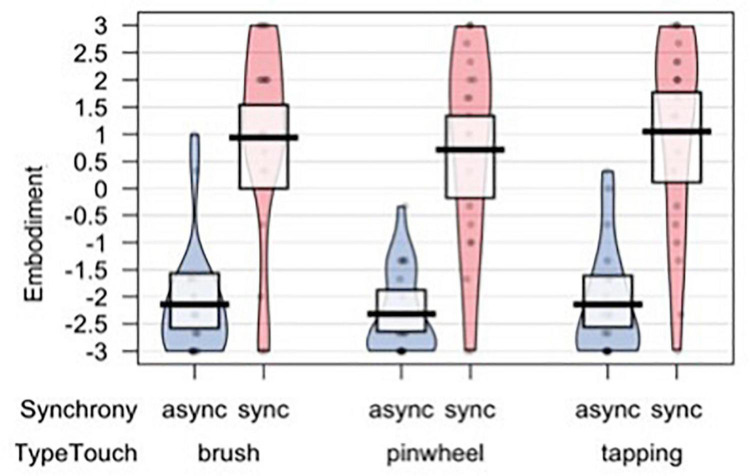
Embodiment score measuring the subjective illusion experience.

To assess subjective illusion experience we analyzed an embodiment score based on the first three items of the Embodiment Questionnaire, which was the dependent variable of all models. A set of four nested mixed-effects models were tested, including the same factors used to analyze subjective evaluations of tactile experience. The likelihood ratio test showed that Model 1 was the best at predicting the collected data and included the factor Synchrony (descriptive statistics and model comparison are reported in Supplementary Tables 9,10). The model explained 53% of the variance. A main effect of Synchrony emerged [χ^2^(1) = 230.7, *p* < 0.001; Figure 6]. Notably, the Model 1 remained the best fitting model even when including the subjective evaluations of the tactile experience and SCLs as predictors (see Supplementary Table 11), indicating that the visual-tactile synchrony is the strongest factor driving the subjective illusory experience.

### Proprioceptive Drift

**FIGURE 7:**
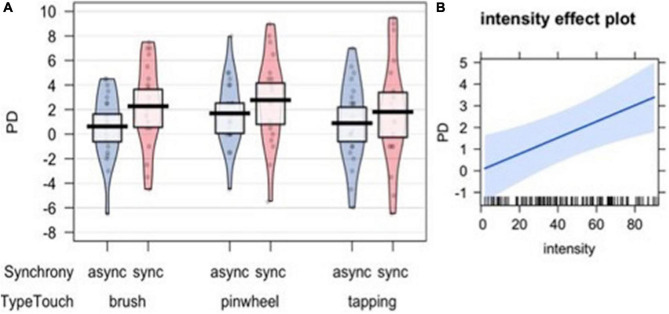
Proprioceptive Drift (PD) measuring the shift in the localization of participants’ own hand after the visuo-tactile induction of the illusion **(A)**. The right panel represents the effect of subjective intensity in driving the PD **(B)**.

In order to analyze whether the illusion resulted in a shift in the proprioceptive perceived position of the participants’ hand, we calculated the difference score between post-stimulation and pre-stimulation pointing (proprioceptive drift – PD). A set of four nested mixed-effects models were tested including the same factors used to analyze subjective embodiment. The likelihood ratio test showed that Model 1 was the best at predicting the collected data and included the factor Synchrony (descriptive statistics and model comparison are reported in Supplementary Tables 12,13). The model explained 3% of the variance. The main effect of Synchrony emerged [χ^2^(1) = 9.07, *p* = 0.003; Figure 7A].

Interestingly, when including subjective evaluations of the tactile experiences (pleasantness – Model 4, painful sensation – Model 5, intensity – Model 6) and the SCL (Model 7) as predictors, the likelihood ratio test showed that Model 6 was the best at predicting the collected data (see Supplementary Table 14). The model explained 12% of the variance. Anova revealed a main effect of Synchrony [χ^2^(1) = 4.01, *p* = 0.045] and a main effect of Intensity [χ^2^(1) = 9.11, *p* = 0.003], indicating that the subjective perception of the tactile experience was a significant factor in modulating the proprioceptive shift (Figure 7B).

In addition, six simple *t*-tests comparing the PD with the null level (zero) were separately performed for each experimental condition in order to explore whether the visual-tactile stimulation elicited a significant shift from the initial localization of the hand. The results revealed that only when the tactile stimulation on the participants’ real hand was performed with a brush or with a pinwheel in synchrony with the stimulation on the rubber hand, did participants show a significant change in the proprioceptive position of their own hand [*t*(20) = 3.04, *p* = 0.006, Cohen’s *d* = 0.66 for Brush synchronous and *t*(20) = 3.54, *p* = 0.002, Cohen’s *d* = 0.77 for Pinwheel synchronous; *p*-value adjusted for multiple comparisons using Bonferroni correction, *p* < 0.008] (Table 1).

**TABLE 1:** Descriptive statistics for each type of visual-tactile stimulation (mean and standard deviation of the PD) and simple *t*-test comparing the PD scores with the null level.

	Type of visual-tactile stimulation					
	Brush sync	Brush async	Pinwheel sync	Pinwheel async	Tapping sync	Tapping async
PD	2.27 (3.42)	0.63 (2.75)	2.77 (3.59)	1.69 (2.79)	1.81 (4.27)	0.89 (3.34)
Simple t-test (null level)	*t* = 3.04 *p* = 0.006 Cohen’s *d* = 0.66	*t* = 1.05 *p* = 0.305 Cohen’s *d* = 0.23	*t* = 3.54 *p* = 0.002 Cohen’s *d* = 0.77	*t* = 2.78 *p* = 0.012 Cohen’s *d* = 0.61	*t* = 1.94 *p* = 0.066 Cohen’s *d* = 0.27	*t* = 1.22 *p* = 0.235 Cohen’s *d* = 0.42

*p-value adjusted for multiple comparisons using Bonferroni correction, p < 0.008.*

## Discussion

The body is the means by which we perceive and interact with the surrounding environment. Thus, investigating how we integrate different sensory signals into the unified perception of our own body is of particular relevance for the understanding of body self-awareness, which represents a starting point for sensory cognitive and social processes. The RHI provides a well-established method for studying the modulation of the bodily self, based on the integration of multisensory information from vision and touch. Previous studies have shown some evidence that receiving a pleasant affective touch (3 cm/s caress-like CT-mediated touch) during the paradigm elicits a stronger illusion (Crucianelli et al., 2013, 2018; Lloyd et al., 2013; van Stralen et al., 2014). This effect was hypothesized to be driven by the activation of interoceptive processes linked to CT- afferents (Tsakiris et al., 2007; Crucianelli et al., 2013, 2018).

In the current study we aimed to investigate how different qualities of touch, involving or not interoceptive processes, determine the strength of the RHI. Specifically, we compared the effects of affective touch (pleasure) to an unpleasant touch (pain), which involves, as well, interoceptive processes while having an opposite hedonic valence (positive vs. negative). Moreover, we compared both these two conditions to a control neutral touch with no involvement of interoceptive processes and expected not to have a specific valence. On one side, it was hypothesized that affective touch and the related activation of the CT system have a unique contribution in modulating bodily awareness, and therefore in determining a stronger RHI. In turn, this may indicate the importance of a positive affective valence of touch in modulating the strength of the illusion. On the other hand, it is also possible that the effect previously found can be generalizable to other forms of interoceptive activation, such as pain (unpleasant touch). If this were the case, results would indicate the importance of an interoceptive involvement in strengthening the illusion, regardless of the related affective valence. Moreover, to disentangle possible mechanisms modulating the strength of the illusion, we assessed the subjective affective experience of the tactile stimulation considering two dimensions: the valence of the stimulation (level of pleasantness and sensation of pain) and the perceived intensity (Russell, 1980; Posner et al., 2005; Rubin and Talarico, 2009). As a physiological correlate of the intensity of the affective activation we also measured levels of arousal in terms of skin conductance levels (SCLs) during the visuo-tactile stimulations.

Preliminary analyses on the subjective evaluations of the visuo-tactile experiences suggest that the three types of touch selected were effective in evoking the expected perceptual sensations: stroking with a brush was reported as the most pleasant condition, while rubbing with a pinwheel was considered the most intense and painful sensation. It is important to notice how the tactile stimulations were successful in target specific subjective experiences, suggesting that pleasantness and painfulness are two separate aspects of subjective perception. Specifically, stroking with a brush was the only stimulation that evoked a sense of pleasantness, while touch with the pinwheel was perceived as a painful sensation even if it was not necessarily unpleasant. Indeed, we carefully chose the painful condition in order to elicit a subjective experience related to pain without causing any harm or discomfort to participants. In line with self-reported levels of intensity, the mean SCL during rubbing with a pinwheel was higher compared to the other tactile conditions, although statistical significance was not reached. Interestingly, the synchrony between the touch felt on the real hand and seen on the rubber hand has an impact on the subjective experience of the tactile sensation. Indeed, in line with previous findings (Schütz-Bosbach et al., 2009), participants felt the brush stimulation more pleasant during synchronous than asynchronous stimulation participants reported synchronous visual-tactile stimulations to be more intense than asynchronous, irrespectively of the type of touch. In other words, participants perceived as more intense the tactile stimulation when they were more likely to perceive the illusion (i.e., in the synchronous condition). Therefore, we speculate that the level of activation (i.e., intensity) perceived by participants is associated with occurred multisensory integration and interoceptive activation.

With regard to the main outcomes of the study, our results are consistent with previous findings based on the RHI. Indeed, both measures of subjective embodiment and proprioceptive drift have been found to be sensitive to the synchronicity of visual-tactile stimulation, indicating that spatial-temporal congruency of multisensory signals is the main driver of the illusion. The different types of touch were found not to significantly influence the strength of the illusion. However, the perceived intensity of the stimulation predicted the mis-location of participants’ hand as closer to the rubber hand (proprioceptive drift). Moreover, participants showed a significant shift away from the initial proprioceptive felt position of their own hand toward the rubber hand during synchronous brush stroking and pinwheel rubbing. Importantly, these two conditions have been reported also to be more intense compared to tapping with a stick and are believed to involve interoceptive processing. Taken together, these results suggest that tactile stimulations associated with interoceptive sensations, such as affective touch and pain, may critically contribute to the proprioceptive localization of the body. Previous evidence suggests that separate mechanisms of multisensory integration underlie the spatial update and feeling of ownership (Rohde et al., 2011). More specifically, proprioceptive drift has been suggested to rely on visuo-proprioceptive integration that is inhibited by prolonged asynchronous stroking (Rohde et al., 2011). Thus, it is possible to speculate that interoceptive sensations play an essential role in modulating visual-proprioceptive integration processes, impacting the formation of a bias toward the visual position of the hand. Moreover, these results suggest the importance of the subjective affective experience in terms of intensity in influencing the strength of the illusion. As the conditions involving interoceptive processes were also rated as more intense, and as intensity across conditions was found to predict the proprioceptive drift, we speculate that the reported level of intensity represents the subjectively perceived correlate of an interoceptive activation.

Accordingly, as shown above, participants perceived as more intense also the synchronous condition of the RHI, during which multisensory integration and interoceptive activation are more likely to occur. This is in line with previous research that underlined the link between interoceptive processes and subjective affective experiences. Indeed, interoception has been proposed to be linked to bodily arousal responses and affective/emotional states (Herbert et al., 2007). Notably, interpersonal differences in sensitivity to internal bodily responses (i.e., interoception) reflect variations in the reported intensity of emotional experience (Critchley et al., 2004). At the neural level, the insula and the anterior cingulate cortex (part of the limbic system) are suggested to play important roles for linking interoceptive processing and conscious perception of subjective feeling states (Craig, 2002). Therefore, suggesting that the limbic system may represent the neurobiological substrate where interoceptive information is registered and integrated with other sensory and psychological information to form a coherent percept. While this remains a tentative speculation, future research should investigate this topic, focusing on subjective levels of activation (i.e., intensity) as a correlate of interoceptive sensitivity, which may play a crucial role in modulating body related visuo-tactile integration processing. In corroboration to this interpretation, a virtual reality study (Fusaro et al., 2016) presenting participants with needle penetrating (pain), caress (pleasure), or ball touching (neutral) the hand of an avatar seen from a first vs. third person perspective showed that pain and pleasure were experienced as more salient than neutral at both a subjective and a physiological level. More specifically, results of the study suggested that the perceived intensity of the observed stimuli was maximal for pain, intermediate for pleasure, and minimal for the neutral touch, and that observation of painful stimuli induced a higher sense of ownership compared to the other conditions (Fusaro et al., 2016). Notably, a replication of this study in which the human kinematics were implemented to create a more naturalistic caress-like stimulation, found increased ratings of embodiment in both the pain and pleasure conditions in comparison to the neutral one (Fusaro et al., 2019). Although these studies had a different design from the current one by analyzing vicarious touch to virtual painful and pleasant stimuli, results seem to be in line with the results of our study.

The multisensory representation of the body is an essential prerequisite for differentiating and comparing oneself with others (Meltzoff et al., 2019), which in turns lays the foundations for social understanding and interactions (Brozzoli et al., 2013). Indeed, interoceptive information derived from first-person experiences can serve as a neurophysiological and emotional framework for understanding the actions, goals, and psychological states of others (Ropar et al., 2018), through a process of self-other identification that first takes place in the bodily domain (Maister et al., 2017). Thus, atypical interoception and multisensory integration could affect the development of body awareness and the malleability of one’s own bodily representation, impacting higher-order social and cognitive processes, including the understanding of others’ action and emotions (Ropar et al., 2018). This is particularly evident in psychopathology, for example in the contexts of eating disorders (EDs; Teaford et al., 2021). Indeed, ED patients present with profound alterations in body perception that include deficits in interoception and multisensory integration as well as abnormalities in body representation (Crucianelli et al., 2020; Sacchetti et al., 2021; Teaford et al., 2021). Interestingly, these alterations in body perception have been found to be coupled with higher-order psycho-social impairments and specifically with difficulties in understanding others’ behaviors and emotions (Caglar-Nazali et al., 2014; Bora and Köse, 2016). This clinical evidence confirms the posit that interoception and multisensory integration shape the sense of body ownership with important implications for socio-emotional processing.

However, the current study presents with some limitations that are worth mentioning. First of all, it should be noted that the sample size was fairly small due to the fact that collection of data was interrupted by COVID pandemic. At the physiological level, arousal was measured in terms of SCL, which typically reflects autonomic responses. However, multi-recording of additional physiological measures would provide a more extensive understanding of physiological mechanisms underpinning the RHI. Specifically, facial electromyography can be used as an implicit measure of affective valence, considering the activation of the zygomaticus muscle (smile expression) that reflects positive affect and activation of the corrugator muscle (frown expression) that reflects negative affect (Pawling et al., 2017). Moreover, heart rate changes have also been used as an index of valence of affective stimuli (Wilson et al., 2020). Thus, future research should consider a multidimensional evaluation of tactile experiences using both subjective ratings and physiological measures to better specify the role of valence and the intensity of the stimulation in influencing the strength of the RHI. Moreover, future investigation should further analyze the link between the subjective intensity of the stimulation and interoceptive processes, with the goal to better understand their role in influencing multisensory integration. A possible way to address this issue would be to replicate a design similar to this study with different types of touch while modulating or having control over the strength of the stimulation.

Lastly, it should be noted that different individuals may perform differently during the RHI due to differences in personality traits, prior experiences, and attentional mechanisms. For example, musicians have been found to be less susceptible to the RHI possibly due to their prior expertise in motor coordination (Pyasik et al., 2019). Similarly, personality characteristics such as negative body image, schizotypal traits, empathy and suggestibility (i.e., the propensity to be influenced by external factors) have been found to modulate the intensity of the illusion (Peled et al., 2000; Mussap and Salton, 2006; Asai et al., 2011; Eshkevari et al., 2012; Lush et al., 2020). In a similar way, individual differences in interoceptive sensitivity (i.e., the propensity to be interoceptively cognizant) may have an impact in determining the strength of the illusion (Tsakiris et al., 2011; Suzuki et al., 2013). Likewise, it could be worth considering individuals’ tendency to focus their attention on certain incoming stimuli rather than others, and for example on stimuli perceived as threats to the integrity of the body such as painful stimuli (Legrain et al., 2011). In order to address these issues, some exploratory analyses were run between individual tendencies, measured through questionnaires (BAQ, BVAQ, and STQ), and experimental measures (subjective evaluation of touch, embodiment scores, and proprioceptive drift). However, given the small sample size it was not possible to discuss them and we decided to report them in the Supplementary Materials section. Future studies with larger samples should therefore consider the influence of individual characteristics and personality traits, especially in terms of interoceptive sensitivity, in modulating the RHI and the effects of different types of touch on the RHI.

To conclude, here we showed, that pleasant and painful tactile stimulations, both involving interoceptive processes while having opposite hedonic valence (positive vs. negative), can both induce a drift in the proprioceptive location of the hand during the RHI (proprioceptive drift). Interestingly, these two tactile stimulations were also perceived as more intense by participants. At the same time, intensity of the tactile stimulation was found to predict a stronger proprioceptive drift across the different conditions. We propose that interoceptive information, regardless of the valence of the stimuli (positive and negative) are subjectively perceived as more intense and, through the activation of the limbic system, enhance multisensory integration during the RHI.

## Data Availability Statement

The raw data supporting the conclusions of this article will be made available by the authors, without undue reservation.

## Ethics Statement

The studies involving human participants were reviewed and approved by the Liverpool John Moores University Research Ethics Committee. The patients/participants provided their written informed consent to participate in this study.

## Author Contributions

All authors discussed the project and developed the hypothesis. LD and SS designed the method, prepared the materials, collected and analyzed the data, and prepared the manuscript. All authors reviewed the manuscript and approved the final version of the manuscript.

## Conflict of Interest

The authors declare that the research was conducted in the absence of any commercial or financial relationships that could be construed as a potential conflict of interest.

## Publisher’s Note

All claims expressed in this article are solely those of the authors and do not necessarily represent those of their affiliated organizations, or those of the publisher, the editors and the reviewers. Any product that may be evaluated in this article, or claim that may be made by its manufacturer, is not guaranteed or endorsed by the publisher.
