# Supplementary material for: Does Nice or Nasty Matter? The Intensity of Touch Modulates the Rubber Hand Illusion
Source: Front Psychol. 2022 Jun 13;13:901413. doi: 10.3389/fpsyg.2022.901413 (PMC9234571; doi:10.3389/fpsyg.2022.901413)
Supplement: Supplementary file 1 [file Data_Sheet_1.docx]

**Supplementary Materials**

**Supplementary methods**

**Body Awareness Questionnaire (BAQ; Shields et al., 1989)**

The BAQ is a 18-item self-report inventory investigating participants’ awareness in regard to their bodily states. Participants are asked to rate on a 7 point Likert scale how sensitive they usually are to normal, non-emotive body processes (e.g. thirst, hunger, fatigue, temperature changes). Items are rated from a minimum of 1 (“Not at all true of me”) to a maximum of 7 (Very true of me).

**Pain Awareness and Vigilance Questionnaire (PVAQ; McCracken 1997)**

The PVAQ is a 16-item self-report questionnaire. Items are registered on a six-point scale, ranging from 0 (never) to 5 (always); thus, the total questionnaire scores range between 0 and 80. The PVAQ measures the frequency of self-monitored and self-reported attentional habits with the focus on pain and changes in pain over the past 2 weeks. Previous studies have reported good reliability and validity values for the PVAQ (Roelofs et al, 2002; Kunz et al., 2017).

**Social Touch Questionnaire (STQ; Wilhelm, et al. 2001)**

The STQ is a self-report questionnaire for adolescents and adults measuring behaviors and attitudes towards social touch. It comprises 20 items that are to be answered on a 5-point Likert-scale from 0 (not at all) to 4 (extremely). The items reflect different varieties of social touch, such as touch among friends and family, and touch among strangers, or the liking and disliking of touch in various situations. Previous studies have reported good reliability and validity values for the STQ (Vieira et al, 2016; Lapp & Croy., 2021).

**Supplementary data analyses**

**Pleasantness**

Descriptive Statistics:

|  | **Pleasant Touch**  (stroking with a brush) | **Unpleasant Touch**  (rubbing with a pinwheel) | **Neutral Touch**  (Tapping with a stick) |
| --- | --- | --- | --- |
| **Synchronous** | 4.98 (2.67) | 0.34 (4.01) | 0.83 (1.69) |
| **Asynchronous** | 2.36 (4.39) | 0.99 (3.42) | 0.09 (2.22) |

Table 1. Descriptive statistics (mean and standard deviation) of pleasantness ratings from -10 (very unpleasant) to +10 (very pleasant)

Model comparison:

| **Tested models** | **Variables** | **AIC** | **Delta AIC** | **BIC** | **Marginal R^2^** | **Conditional R^2^** | **χ2** | ***p*** |
| --- | --- | --- | --- | --- | --- | --- | --- | --- |
| Model 0 | Random effect of Participants | 669.55 |  | 678.05 |  | 0.268 |  |  |
| Model 1 | + Synchrony | 668.78 | 1.37 | 680.13 | .016 | 0.285 | 2.765 | .096 |
| Model 2 | + Type of Touch | 638.12 | 31.44 | 655.14 | .182 | 0.480 | 34.660 | < .001 |
| Model 3 | + Synchrony X Type of Touch | 633.22 | 8.40 | 655.91 | .215 | 0.516 | 8.899 | .012 |

Table 2. Comparison between models predicting subjective pleasantness of tactile stimulations. Note that smaller values of AIC and BIC indicate better fitting models.

Both AIC and BIC indices indicate that Model 2 is a good fit. However, based on AIC Model 3, that also included the interaction effect, seems to be slightly better in predicting the collected data. In keeping with a more explorative approach, we select Model 3 for our analyses.


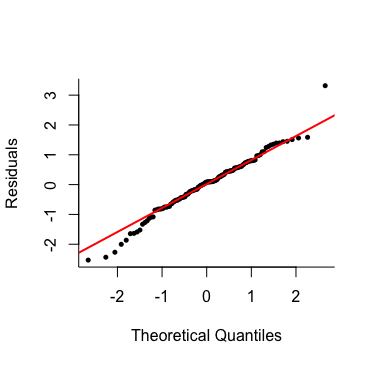


Figure 1. Q-Q plot to control for normality of residuals of plesantness ratings

Analysis of Deviance Table (Type II Wald chisquare tests)

Response: pleasantness

Chisq Df Pr(>Chisq)

Synchrony 4.0394 1 0.04445 *

TypeTouch 42.5693 2 5.704e-10 ***

Synchrony:TypeTouch 8.8452 2 0.01200 *

**Pain**

Descriptive Statistics:

|  | **Pleasant Touch**  (stroking with a brush) | **Unpleasant Touch**  (rubbing with a pinwheel) | **Neutral Touch**  (Tapping with a stick) |
| --- | --- | --- | --- |
| **Synchronous** | 2.80 (6.14) | 28.02 (27.91) | 6.69 (13.08) |
| **Asynchronous** | 6.34 (13.86) | 26.92 (24.08) | 6.27 (10.32) |

Table 3. Descriptive statistics (mean and standard deviation) of pain ratings on a scale from 0 to 100.

Model comparison:

| **Tested models** | **Variables** | **AIC** | **Delta AIC** | **BIC** | **Marginal R^2^** | **Conditional R^2^** | **χ2** | ***p*** |
| --- | --- | --- | --- | --- | --- | --- | --- | --- |
| Model 0 | Random effect of Participants | 1103.0 |  | 1111.5 |  | 0.304 |  |  |
| Model 1 | + Synchrony | 1104.9 | 2.10 | 1116.3 | < .001 | 0.302 | 0.049 | .825 |
| Model 2 | + Type of Touch | 1046.3 | 65.45 | 1063.4 | .260 | 0.610 | 62.573 | < .001 |
| Model 3 | + Synchrony X Type of Touch | 1049.5 | 7.08 | 1072.2 | .261 | 0.608 | 0.835 | .659 |

Table 4. Comparison between models predicting subjective painful sensation during tactile stimulations. Note that smaller values of AIC and BIC indicate better fitting models.

Both AIC and BIC indices point to Model 2 as the best fitting model.


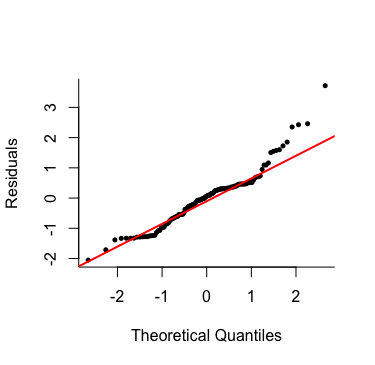


Figure 2. Q-Q plot to control for normality of residuals of pain ratings

Analysis of Deviance Table (Type II Wald chisquare tests)

Response: painful

Chisq Df Pr(>Chisq)

Synchrony 0.0862 1 0.769

TypeTouch 83.1024 2 <2e-16 ***

**Intensity**

Descriptive Statistics:

|  | **Pleasant Touch**  (stroking with a brush) | **Unpleasant Touch**  (rubbing with a pinwheel) | **Neutral Touch**  (Tapping with a stick) |
| --- | --- | --- | --- |
| **Synchronous** | 47.62 (25.87) | 58.85 (16.72) | 38.90 (23.58) |
| **Asynchronous** | 38.15 (24.83) | 54.59 (19.49) | 26.39 (21.44) |

Table 5. Descriptive statistics (mean and standard deviation) of intensity ratings on a scale from 0 to 100.

Model comparison:

| **Tested models** | **Variables** | **AIC** | **Delta AIC** | **BIC** | **Marginal R^2^** | **Conditional R^2^** | **χ2** | ***p*** |
| --- | --- | --- | --- | --- | --- | --- | --- | --- |
| Model 0 | Random effect of Participants | 1141.8 |  | 1163.2 |  | 0.257 |  |  |
| Model 1 | + Synchrony | 1138.2 | 7.89 | 1162.5 | .031 | 0.293 | 5.551 | .018 |
| Model 2 | + Type of Touch | 1108.3 | 38.19 | 1138.3 | .193 | 0.482 | 33.937 | < .001 |
| Model 3 | + Synchrony X Type of Touch | 1111.1 | 8.53 | 1146.7 | .196 | 0.482 | 1.200 | .549 |

Table 6. Comparison between models predicting subjective intensity of tactile stimulations. Note that smaller values of AIC and BIC indicate better fitting models.

Both AIC and BIC indices point to Model 2 as the best fitting model.


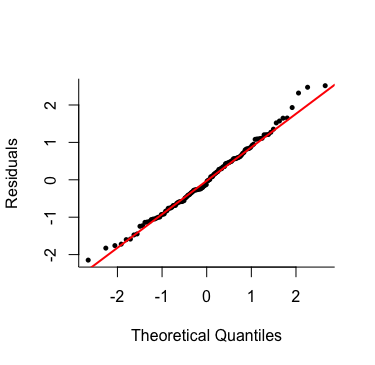


Figure 3. Q-Q plot to control for normality of residuals of intensity ratings

Analysis of Deviance Table (Type II Wald chisquare tests)

Response: intensity

Chisq Df Pr(>Chisq)

Synchrony 7.6505 1 0.005676 **

TypeTouch 38.9190 2 3.539e-09 ***

**SCL**

Descriptive Statistics:

|  | **Pleasant Touch**  (stroking with a brush) | **Unpleasant Touch**  (rubbing with a pinwheel) | **Neutral Touch**  (Tapping with a stick) |
| --- | --- | --- | --- |
| **Synchronous** | 0.14 (1.50) | -0.32 (1.60) | -0.48 (1.57) |
| **Asynchronous** | -0.28 (1.23) | 0.22 (1.65) | -0.59 (1.65) |

Table 7. Descriptive statistics (mean and standard deviation) of modulation of SCL during visuo-tactile stimulation compared to pre-stimulus baseline.

Model comparison:

| **Tested models** | **Variables** | **AIC** | **Delta AIC** | **BIC** | **Marginal R^2^** | **Conditional R^2^** | **χ2** | ***p*** |
| --- | --- | --- | --- | --- | --- | --- | --- | --- |
| Model 0 | Random effect of Participants | 452.14 |  | 460.65 |  | 0.319 |  |  |
| Model 1 | + Synchrony | 454.14 | -3.11 | 465.49 | < .001 | 0.316 | <0.001 | .986 |
| Model 2 | + Type of Touch | 454.11 | -1.77 | 471.13 | .021 | 0.336 | 4.032 | .133 |
| Model 3 | + Synchrony X Type of Touch | 454.80 | 0.20 | 477.49 | .038 | 0.351 | 3.308 | .191 |
| Model 4 | + Pleasantness | 452.36 | -2.17 | 477.89 | .071 | 0.368 | 4.446 | .035 |
| Model 5 | + Pain | 451.27 | -6.69 | 479.63 | .097 | 0.401 | 3.093 | .078 |
| Model 6 | + Intensity | 452.39 | -9.32 | 483.59 | .099 | 0.404 | 0.874 | .350 |

Table 8. Comparison between models predicting SCL. Note that smaller values of AIC and BIC indicate better fitting models.

Both AIC and BIC indices suggest that all models considered do not explain much more variability compared to the null model. However, based on AIC Model 5 seems to be slightly better in predicting the collected data. In keeping with a more explorative approach, we select Model 5 for our analyses.


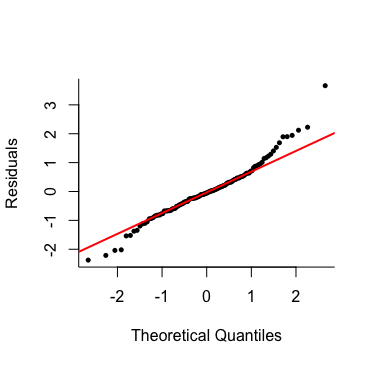


Figure 4. Q-Q plot to control for normality of residuals of SCL

Analysis of Deviance Table (Type II Wald chisquare tests)

Response: SCL

Chisq Df Pr(>Chisq)

Synchrony 0.1446 1 0.70373

TypeTouch 5.9997 2 0.04979 *

pleasantness 1.9158 1 0.16633

painful 2.9611 1 0.08529 .

Synchrony:TypeTouch 1.7264 2 0.42180

**Embodiment**

Descriptive Statistics:

|  | **Pleasant Touch**  (stroking with a brush) | **Unpleasant Touch**  (rubbing with a pinwheel) | **Neutral Touch**  (Tapping with a stick) |
| --- | --- | --- | --- |
| **Synchronous** | 0.94 (1.81) | 0.71 (1.87) | 1.05 (1.84) |
| **Asynchronous** | -2.14 (1.06) | -2.31 (0.79) | -2.14 (1.02) |

Table 9. Descriptive statistics (mean and standard deviation) of Embodiment Questionnaire scores.

Model comparison:

| **Tested models** | **Variables** | **AIC** | **Delta AIC** | **BIC** | **Marginal R^2^** | **Conditional R^2^** | **χ2** | ***p*** |
| --- | --- | --- | --- | --- | --- | --- | --- | --- |
| Model 0 | Random effect of Participants | 551.26 |  | 559.77 |  | 0.075 |  |  |
| Model 1 | + Synchrony | 430.54 | 119.38 | 441.88 | .532 | 0.712 | 122.725 | < .001 |
| Model 2 | + Type of Touch | 433.38 | -5.02 | 450.40 | .532 | 0.711 | 1.154 | .562 |
| Model 3 | + Synchrony X Type of Touch | 437.27 | -3.24 | 459.96 | .530 | 0.707 | 0.109 | .947 |

Table 10. Comparison between models predicting embodiment over the rubber hand. Note that smaller values of AIC and BIC indicate better fitting models.

Both AIC and BIC indices point to Model 1 as the best fitting model.

| **Tested models** | **Variables** | **AIC** | **Delta AIC** | **BIC** | **Marginal R^2^** | **Conditional R^2^** | **χ2** | ***p*** |
| --- | --- | --- | --- | --- | --- | --- | --- | --- |
| Model 0 | Random effect of Participants | 551.26 |  | 559.77 |  | 0.075 |  |  |
| Model 1 | + Synchrony | 430.54 | 119.38 | 441.88 | .532 | 0.712 | 122.725 | < .001 |
| Model 2 | + Type of Touch | 433.38 | -5.02 | 450.40 | .532 | 0.711 | 1.154 | .562 |
| Model 3 | + Synchrony X Type of Touch | 437.27 | -3.24 | 459.96 | .530 | 0.707 | 0.109 | .947 |
| Model 4 | + Pleasantness | 438.39 | -5.67 | 463.92 | .524 | 0.715 | 0.884 | .347 |
| Model 5 | + Pain | 433.00 | -2.78 | 461.37 | .542 | 0.736 | 7.386 | .007 |
| Model 6 | + Intensity | 432.68 | -8.13 | 463.88 | .553 | 0.734 | 2.321 | .128 |
| Model 7 | + SCL | 434.70 | -5.07 | 468.70 | .551 | 0.733 | 0.012 | .912 |

Table 11. Comparison between models predicting embodiment over the rubber hand, including the subjective evaluations of tactile experiences and SCL. Note that smaller values of AIC and BIC indicate better fitting models.

Both AIC and BIC indices point to Model 2 as the best fitting model.


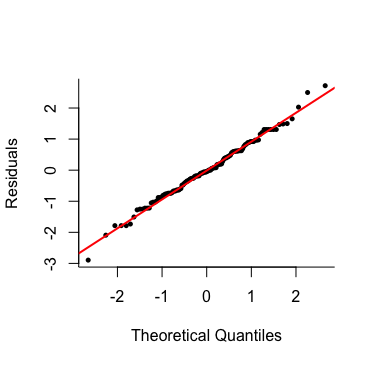


Figure 5. Q-Q plot to control for normality of residuals of embodiment

Analysis of Deviance Table (Type II Wald chisquare tests)

Response: Embodiment_Score1

Chisq Df Pr(>Chisq)

Synchrony 230.69 1 < 2.2e-16 ***

**Proprioceptive Drift**

Descriptive Statistics:

|  | **Pleasant Touch**  (stroking with a brush) | **Unpleasant Touch**  (rubbing with a pinwheel) | **Neutral Touch**  (Tapping with a stick) |
| --- | --- | --- | --- |
| **Synchronous** | 2.27 (3.42) | 2.77 (3.59) | 1.81 (4.27) |
| **Asynchronous** | 0.63 (2.75) | 1.69 (2.79) | 0.89 (3.34) |

Table 12. Descriptive statistics (mean and standard deviation) of Proprioceptive Drift.

Model comparison:

| **Tested models** | **Variables** | **AIC** | **Delta AIC** | **BIC** | **Marginal R^2^** | **Conditional R^2^** | **χ2** | ***p*** |
| --- | --- | --- | --- | --- | --- | --- | --- | --- |
| Model 0 | Random effect of Participants | 621.23 |  | 629.74 |  | 0.537 |  |  |
| Model 1 | + Synchrony | 614.46 | 6.79 | 625.80 | .031 | 0.571 | 8.778 | .003 |
| Model 2 | + Type of Touch | 614.54 | 0.39 | 631.55 | .044 | 0.581 | 3.920 | .141 |
| Model 3 | + Synchrony X Type of Touch | 617.91 | -0.08 | 640.60 | .046 | 0.579 | 0.624 | .732 |

Table 13. Comparison between models predicting proprioceptive drift. Note that smaller values of AIC and BIC indicate better fitting models.

Both AIC and BIC indices point to Model 2 as the best fitting model.

| **Tested models** | **Variables** | **AIC** | **Delta AIC** | **BIC** | **Marginal R^2^** | **Conditional R^2^** | **χ2** | ***p*** |
| --- | --- | --- | --- | --- | --- | --- | --- | --- |
| Model 0 | Random effect of Participants | 621.23 |  | 629.74 |  | 0.537 |  |  |
| Model 1 | + Synchrony | 614.46 | 6.79 | 625.80 | .031 | 0.571 | 8.778 | .003 |
| Model 2 | + Type of Touch | 614.54 | 0.39 | 631.55 | .044 | 0.581 | 3.920 | .141 |
| Model 3 | + Synchrony X Type of Touch | 617.91 | -0.08 | 640.60 | .046 | 0.579 | 0.624 | .732 |
| Model 4 | + Pleasantness | 616.10 | -5.67 | 641.63 | .524 | 0.593 | 3.814 | .051 |
| Model 5 | + Pain | 614.60 | -2.78 | 642.96 | .542 | 0.620 | 3.501 | .061 |
| Model 6 | + Intensity | 607.15 | -8.13 | 638.35 | .553 | 0.636 | 9.451 | .002 |
| Model 7 | + SCL | 608.11 | -5.07 | 642.15 | .551 | 0.644 | 1.035 | .309 |

Table 14. Comparison between models predicting proprioceptive drift, including the subjective evaluations of tactile experiences and SCL. Note that smaller values of AIC and BIC indicate better fitting models.

Both AIC and BIC indices indicate that Model 1 is a good fit. However, based on AIC Model 6, which also included subjective ratings, seems to be slightly better in predicting the collected data. In keeping with a more explorative approach, we select Model 6 for our analyses.


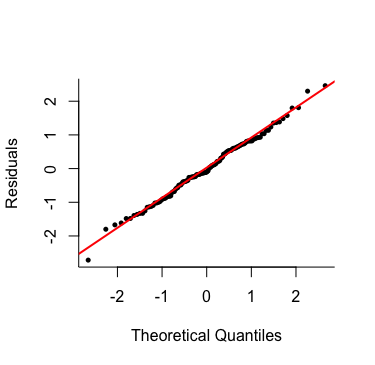


Figure 6. Q-Q plot to control for normality of residuals of proprioceptive drift

Analysis of Deviance Table (Type II Wald chi square tests)

Response: PD

Chisq Df Pr(>Chisq)

Synchrony 4.0107 1 0.045212 *

TypeTouch 1.5147 2 0.468898

pleasantness 1.9002 1 0.168060

painful 0.6652 1 0.414730

intensity 9.1139 1 0.002537 **

Synchrony:TypeTouch 0.6528 2 0.721523

**Correlational Analyses**

In order to explore whether individual differences may have influenced the subjective perception of tactile stimulation, we performed some correlational analysis between individual tendencies to be aware of internal bodily signals (BAQ), to focus on pain sensations (PVAQ) and to show different attitudes towards social touch (STQ) and the mean subjective ratings of pleasantness, pain and intensity of tactile stimulations (Figure 7). Although statistical significance was not reached, it is worthy to mention that BAQ scores showed a trend for a negative correlation with pleasantness (r= -0.28, *p*= .225) and a positive correlation with pain (r= 0.30, *p*= .187). Moreover, PVAQ scores showed a trend for a positive correlation with intensity (r= 0.32, *p*= .159; Tables 15 and 16).


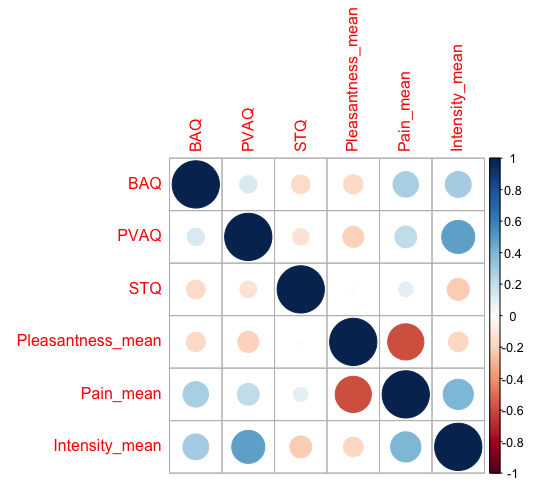


Figure 7. Plots of correlational matrices between questionnaire scores and the mean values of subjective ratings.

|  | BAQ | PVAQ | STQ | Pleasantness  mean | Pain  mean | Intensity  mean |
| --- | --- | --- | --- | --- | --- | --- |
| BAQ | 1.00 | 0.13 | -0.16 | -0.17 | 0.29 | 0.30 |
| PVAQ | 0.13 | 1.00 | -0.12 | -0.20 | 0.21 | 0.49 |
| STQ | -0.16 | -0.12 | 1.00 | 0.01 | 0.09 | -0.22 |
| Pleasantness_mean | -0.17 | -0.20 | 0.01 | 1.00 | -0.59 | -0.17 |
| Pain_mean | 0.29 | 0.21 | 0.09 | -0.59 | 1.00 | 0.41 |
| Intensity_mean | 0.30 | 0.49 | -0.22 | -0.17 | 0.41 | 1.00 |

Table 15 Matrix of correlation coefficients of correlations between questionnaire scores and the mean values of subjective ratings

|  | BAQ | PVAQ | STQ | Pleasantness  mean | Pain  mean | Intensity  mean |
| --- | --- | --- | --- | --- | --- | --- |
| BAQ |  | 0.5657 | 0.5000 | 0.4693 | 0.2023 | 0.1876 |
| PVAQ | 0.5657 |  | 0.5893 | 0.3914 | 0.3548 | 0.0242 |
| STQ | 0.5000 | 0.5893 |  | 0.9534 | 0.6860 | 0.3471 |
| Pleasantness_mean | 0.4693 | 0.3914 | 0.9534 |  | 0.0053 | 0.4507 |
| Pain_mean | 0.2023 | 0.3548 | 0.6860 | 0.0053 |  | 0.0662 |
| Intensity_mean | 0.1876 | 0.0242 | 0.3471 | 0.4507 | 0.0662 |  |

Table 16 Matrix of p-values of correlations between questionnaire scores and the mean values of subjective ratings

More specifically, we also asked whether individual tendencies might be related to the degree of differentiation between tactile conditions. Therefore we calculate a differential score between Brush (pleasant condition) and Pinwheel (unpleasant condition; Figure 8). The results revealed a that participants with higher BAQ and PVAQ scores differentiate more between brush stroking and pinwheel in terms of pleasantness (BAQ: r= 0.35, *p*=.122; PVAQ: r= 0.48, *p*= .027) and pain (r= -0.32, *p*=.153; PVAQ: r= -0.32, *p*= .151). However the two questionnaires differed in relation to intensity scores: participants with higher BAQ scores reported rubbing with a pinwheel as more intense (r= -0.31, *p*= .169), while participants with higher PAVQ scores reported brushing with a brush as more intense (r= 0.30, *p*= .181; Tables 17 and 18).


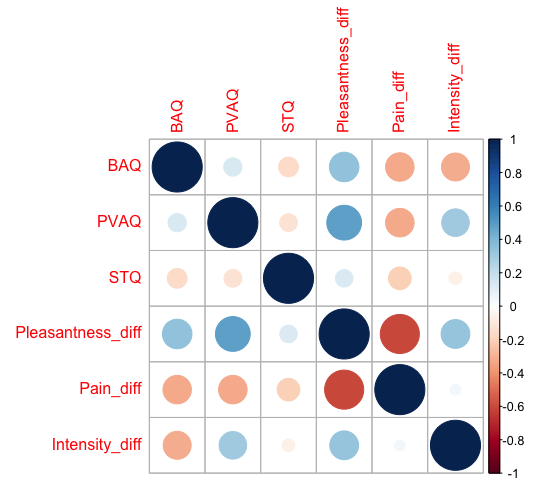


Figure 8. Plots of correlational matrices between questionnaire scores and the differential values (brush - pinwheel).

|  | BAQ | PVAQ | STQ | Pleasantness  mean | Pain  mean | Intensity  mean |
| --- | --- | --- | --- | --- | --- | --- |
| BAQ | 1.00 | 0.13 | -0.16 | 0.35 | -0.32 | -0.31 |
| PVAQ | 0.13 | 1.00 | -0.12 | 0.48 | -0.32 | 0.30 |
| STQ | -0.16 | -0.12 | 1.00 | 0.12 | -0.20 | -0.07 |
| Pleasantness_mean | 0.35 | 0.48 | 0.12 | 1.00 | -0.61 | 0.33 |
| Pain_mean | -0.32 | -0.32 | -0.20 | -0.61 | 1.00 | 0.05 |
| Intensity_mean | -0.31 | 0.30 | -0.07 | 0.33 | 0.05 | 1.00 |

Table 17 Matrix of correlation coefficients of correlations between questionnaire scores and the differential values (brush - pinwheel).

|  | BAQ | PVAQ | STQ | Pleasantness  mean | Pain  mean | Intensity  mean |
| --- | --- | --- | --- | --- | --- | --- |
| BAQ |  | 0.5657 | .0.5000 | 0.1221 | 0.1535 | 0.1692 |
| PVAQ | 0.5657 |  | 0.5893 | 0.0268 | 0.1514 | 0.1812 |
| STQ | 0.5000 | 0.5893 |  | 0.5975 | 0.3744 | 0.7783 |
| Pleasantness_mean | 0.1221 | 0.0268 | 0.5975 |  | 0.0034 | 0.1438 |
| Pain_mean | 0.1535 | 0.1514 | 0.3744 | 0.0034 |  | 0.8435 |
| Intensity_mean | 0.1692 | 0.1812 | 0.7783 | 0.1438 | 0.8435 |  |

Table 18 Matrix of p-values of correlations between questionnaire scores and the differential values (brush - pinwheel).

Finally, we explore whether these individual tendencies measured through questionnaires were related to susceptibility to RHI illusion. Specifically, we considered the differential score between synchronous and asynchronous stimulation in proprioceptive drift and embodiment for each tactile stimulation (Figure 9). Results suggest a significant positive correlation between BAQ scores and differential PD in the tapping condition (r= 0.30, *p*= .187). This result may indicate that individuals with higher sensibility for normal internal bodily processes, are more sensitive to changes of visual-tactile synchrony in a neutral tactile condition. Although statistical significance was not reached, other correlational trends merit consideration. Individuals with higher BVAQ scores showed higher differential PD in tapping (r= 0.39, *p*= .079) and the pinwheel conditions (r= 0.33, *p*= .150). Moreover, it is worth looking into STQ scores which showed a trend for a negative correlation with differential embodiment scores in the brush condition (r= -0.28, *p*= .214). This indicated that individuals with aversion to social touch (high STQ scores) are less sensitive to modulate the subjective sense of body awareness in relation to synchronous vs asynchronous visual-tactile stimulation when they perceive brush stroking (pleasant condition). Complete correlation matrix of correlation coefficients and associated p-values can be found in Supplementary Materials (Tables 19 and 20).


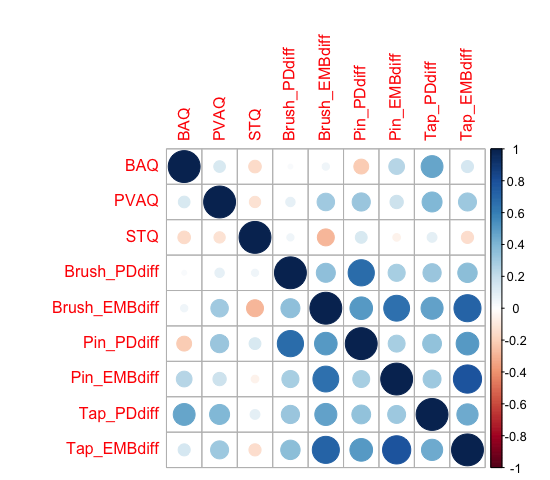


Figure 9. Plot of correlational matrix between questionnaire scores and the differential score (synchronous - asynchronous) of proprioceptive drift and embodiment for each tactile condition.

|  | BAQ | PVAQ | STQ | Brush  PD | Brush  EMB | Pin  PD | Pin  EMB | Tap  PD | Tap  EMB |
| --- | --- | --- | --- | --- | --- | --- | --- | --- | --- |
| BAQ | 1.00 | 0.13 | -0.16 | 0.02 | 0.05 | -0.22 | 0.24 | 0.47 | 0.14 |
| PVAQ | 0.13 | 1.00 | -0.12 | 0.09 | 0.30 | 0.33 | 0.17 | 0.39 | 0.32 |
| STQ | -0.16 | -0.12 | 1.00 | 0.05 | -0.28 | 0.14 | -0.06 | 0.09 | -0.14 |
| Brush_PD | 0.02 | 0.09 | 0.05 | 1.00 | 0.36 | 0.67 | 0.29 | 0.33 | 0.37 |
| Brush_EMB | 0.05 | 0.30 | -0.28 | 0.36 | 1.00 | 0.50 | 0.66 | 0.48 | 0.72 |
| Pin_PD | -0.22 | 0.33 | 0.14 | 0.67 | 0.50 | 1.00 | 0.28 | 0.35 | 0.50 |
| Pin_EMB | 0.24 | 0.17 | -0.06 | 0.29 | 0.66 | 0.28 | 1.00 | 0.32 | 0.78 |
| Tap_PD | 0.47 | 0.39 | 0.09 | 0.33 | 0.48 | 0.35 | 0.32 | 1.00 | 0.44 |
| Tap_EMB | 0.14 | 0.32 | -0.14 | 0.37 | 0.72 | 0.50 | 0.78 | 0.44 | 1.00 |

Table 19 Matrix of correlation coefficients of correlations between questionnaire scores and the differential score (synchronous - asynchronous) of proprioceptive drift and embodiment for each tactile condition.

|  | BAQ | PVAQ | STQ | Brush  PD | Brush  EMB | Pin  PD | Pin  EMB | Tap  PD | Tap  EMB |
| --- | --- | --- | --- | --- | --- | --- | --- | --- | --- |
| BAQ |  | 0.5657 | 0.5000 | 0.9263 | 0.8259 | 0.3457 | 0.2883 | 0.0321 | 0.5379 |
| PVAQ | 0.5657 |  | 0.5893 | 0.7109 | 0.1834 | 0.1503 | 0.4501 | 0.0788 | 0.1574 |
| STQ | 0.5000 | 0.5893 |  | 0.8295 | 0.2141 | 0.5557 | 0.8080 | 0.6861 | 0.5309 |
| Brush_PD | 0.9263 | 0.7109 | 0.8295 |  | 0.1129 | 0.0008 | 0.2059 | 0.1483 | 0.0988 |
| Brush_EMB | 0.8259 | 0.1834 | 0.2141 | 0.1129 |  | 0.0207 | 0.0013 | 0.0284 | 0.0002 |
| Pin_PD | 0.3457 | 0.1503 | 0.5557 | 0.0008 | 0.0207 |  | 0.2154 | 0.1220 | 0.0199 |
| Pin_EMB | 0.2883 | 0.4501 | 0.8080 | 0.2059 | 0.0013 | 0.2154 |  | 0.1616 | 0.0000 |
| Tap_PD | 0.0321 | 0.0788 | 0.6861 | 0.1483 | 0.0284 | 0.1220 | 0.1616 |  | 0.0448 |
| Tap_EMB | 0.5379 | 0.1574 | 0.5309 | 0.0988 | 0.0002 | 0.0199 | 0.0000 | 0.0448 |  |

Table 20 Matrix of p-values of correlations between questionnaire scores and the differential score (synchronous - asynchronous) of proprioceptive drift and embodiment for each tactile condition.
